# Critical reflections on a visit to an inner-city primary health care clinic in Rio de Janeiro

**DOI:** 10.4102/phcfm.v9i1.1420

**Published:** 2017-07-27

**Authors:** Louis S. Jenkins, Marcos A. Goldraich

**Affiliations:** 1 Division of Family Medicine and Primary Care, Faculty of Medicine and Health Sciences, University of Stellenbosch, South Africa; 2 Family and Community Medicine Residency Program, Department of Health, Maria do Socorro Family Clinic, Brazil

## Abstract

**Introduction:**

Brazil and South Africa share many sociodemographic and health features that provide many learning opportunities. Brazil’s national health system, the Sistema Único de Saúde (SUS) prioritises primary health care since 1994, the year democracy came to South Africa. Two family physicians from these countries met in Rocinha favela in Rio de Janeiro, a densely populated area where poverty, danger, drugs, tuberculosis and mental illness are the focus of the health system.

**Maria do Socorro Family Clinic:**

Central to the SUS are the Family Health Teams, consisting of community health workers, nurses, doctors and allied health workers. This clinic in Rocinha has 11 teams, caring for 2700 people each, all visited monthly, preventing illness and promoting health. Patients with mental illness are cared for in a therapeutic residency, with an onsite psychiatrist, psychologist and social worker. The relationships between the health carers and the clinic and the community are collegial and equal, sharing care. Larger than life photos of patients from the community line the walls.

**Training:**

A culture of learning is evident, with 18 family medicine residents, student nurses, a small library and a learning centre at the clinic. Local authorities compensate trainees in family medicine more than traditional specialties.

**Conclusion:**

Brazil has made massive progress in providing universal health coverage over the last 20 years. South Africa, with not too dissimilar challenges, is embarking on this road more recently. The lessons learnt at clinic and community level in this inner-city clinic could be very useful for similar settings in South Africa and other countries.

## Introduction

**FIGURE 1:**
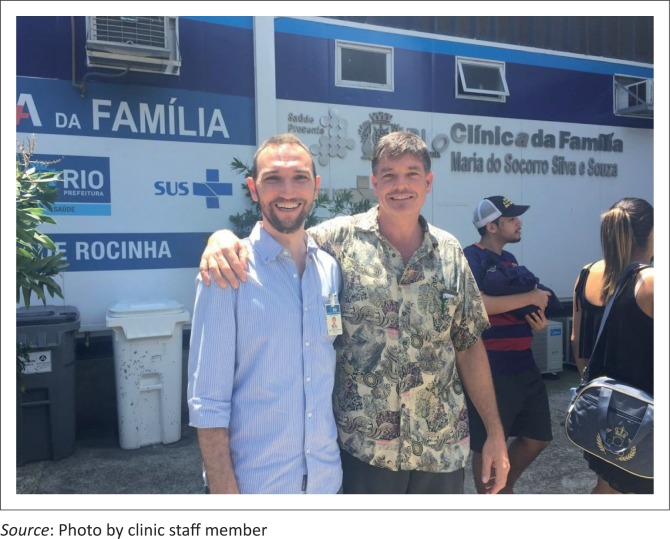
The authors in front of the Maria do Socorro Family Clinic.

South Africa and Brazil share many sociodemographic and health features that provide many learning opportunities. Following the 21st World Conference of Family Doctors held in November 2016 in Rio de Janeiro, the authors, fellow family physicians on either side of the Atlantic Ocean, reconnected. Dr Goldraich is the descendent of German–Polish immigrants during the Second World War. He lives with his wife, also a family doctor and mother of their 1½-year-old daughter, in the city of Rio de Janeiro. He has worked as a clinician and family physician supervisor for the last six years in Rocinha, the largest favela in Rio city, and invited his South African counterpart (first author) to visit the clinic where he works. With a reminder that not all clinics in Brazil look alike, the work happening at this clinic that opened nearly seven years ago is remarkable and needs to be shared with a wider audience (Figure 1).

## Background

Brazil’s national health system, the *Sistema Único de Saúde* (SUS) was introduced in 1994, the year South Africa inaugurated its first democratically elected president, Mr Nelson Mandela. The SUS provides universal health coverage to more than 70% of the country with the world’s fifth largest population.^1,2^ Health expenditure in Brazil amounts to 9.3% of GDP, with out of pocket (private insurance) health expenditure amounting to 57.8% of health expenditure.^2^ South Africa spends 8.6% of the GDP on health, with 60% spent in the private sector for only 16% of the population.^3,4^ In Brazil, 28.9% of the population have private insurance.^5^

Since the introduction of the SUS, prioritising primary health care (PHC), the country has consistently shown an improvement in the major health indices, with life expectancy at birth being 74 years and child deaths under 5 years of age standing at 14 per 1000 live births in 2013.^1,2^ The comparable indicators for South Africa were life expectancy at birth of 60 years and child deaths under 5 years of age standing at 45 per 1000 live births in 2012.^6^

Central to the SUS is a community-based primary care approach with more than 40 000 multi-professional Family Health Teams (FHTs) organised in geographic areas with no overlap or gap between catchment areas.^7^ The most important component of the FHT is the key role of community health agents. Each agent lives in the clinic’s neighbourhood and is assigned to about 150 households or 750 inhabitants in a geographically delineated micro-area within a catchment area. They visit each household within their area at least monthly, irrespective of need or demand, and collect individual- and household-level data.^2^ Their main job is to do home visits, all day long, five days a week. They have only one or two shifts a week in the clinic. Just as family doctors, with continuity of care, they get to know their families, and the visits tend to be shorter, just as are consultations in PHC. Twenty years ago, South Africa published recommendations on the role of community health workers (CHWs). The lessons already then learnt from countries outside South Africa included the realisation that in order for this cadre of worker to reach their expected potential, full community participation in the development of CHW programmes was essential. Small-scale programmes were more successful than national initiatives, mostly because the required financial and policy support had not been forthcoming at a national level. In many countries, planners and communities have had unrealistic expectations of CHWs which has led to disappointment. It is essential for CHWs to have very specific roles and be responsive to the needs of their communities. They need full financial and career pathing support from the health services and the non-governmental organisations (NGOs) working with them. A major challenge for CHWs, working in the grey zone between the community and the rest of the health services, is that of dual accountability. Although they are paid by an NGO, their line management in terms of performance and outcome is the health system.^8^ In Brazil, the presence of CHWs in the FHT is compulsory, as are nurses, doctors and nurse assistants. In order for the city to get federal funding for PHC, the FHT must be complete with all its members. Each team has between 4 and 6 CHWs, and they are usually lay people, living in the community, paid by the city. They get trained in service, with the close supervision of the team’s nurse.

South Africa is implementing a universal health coverage system through the national health insurance (NHI) scheme.^9^ A massive shift from the earlier hospital-based curative approach towards PHC, delivered through the district health system, is taking place. PHC is being re-engineered, with four streams being implemented, namely Municipal Ward-based Primary Health Care Outreach Teams (WBPHCOTs), an Integrated School Health Programme, District Clinical Specialist Teams and contracting of private health practitioners at non-specialist level. The WBPHCOTs are nurse-led, linked to a PHC clinic, and place heavy emphasis on the role of community health care workers. South Africa is slowly recognising that the CHWs are not a cheap solution to health care. Their role has been part of a broader labour context within the country, where the blur of boundaries of employment and volunteerism has been very politicised. The legitimisation of the role of the CHW in policy and service delivery development within the PHC re-engineering model is still being worked out, with many examples of good practice, including Ithemba Lobomi in the Eden district (http://search.info4africa.org.za/Organisation?Id=83854, accessed 15 April 2017) and Chiawelo Community Practice in Soweto (https://afrocp.wordpress.com/chiawelo-cp/, accessed 15 April 2017). These NGOs typically care for pregnant mothers, children and people living with HIV or AIDS, which is largely how the Brazilian CHWs and FHTs started out.^10^ It seems South Africa needs more time and a strong national commitment to strengthen PHC, combined with courageous leadership to address CHW labour issues and re-allocate health funding to grassroots level, thinking beyond the clinic. The NHI white paper describes how the ideal clinic should look, with Operation Phakisa having been launched by the government towards such a model.^11^

The city of Rio de Janeiro is the second largest in Brazil, with 6 million inhabitants, of which 1 million live in 1000 favelas or subnormal clusters (the technical name given by the Brazilian Institute of Geography and Statistics to designated slums and communities with at least 51 households).^12^ Rio was known for having the worst public health system in the country with only 3% coverage of PHC 20 years ago.^2,12^ Since 2009, the city is rapidly increasing the number of FHTs, and now 70% of the city is covered by 1283 FHTs.^13^ One hundred and seventeen new family clinics were built, with 6 to 12 teams based in each clinic. The clinics offer comprehensive PHC, with X-ray, ultrasound, laboratory facilities, minor surgery equipment and dental health teams.^2^

### Rocinha

**FIGURE 2:**
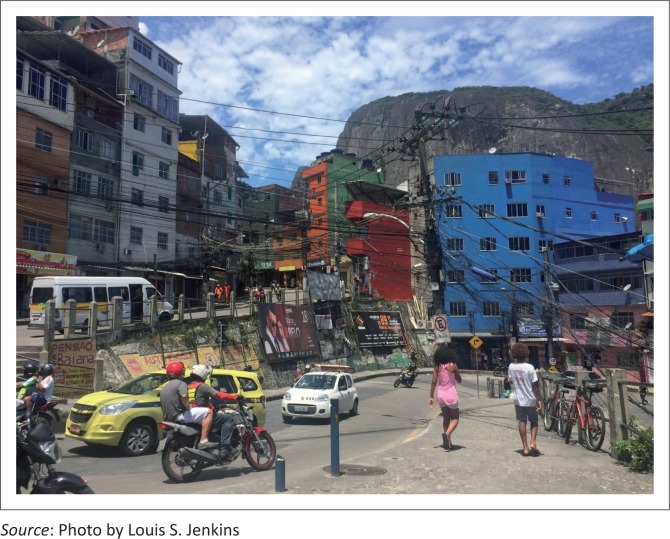
The community of Rocinha, viewed from the entrance to the clinic.

**FIGURE 3:**
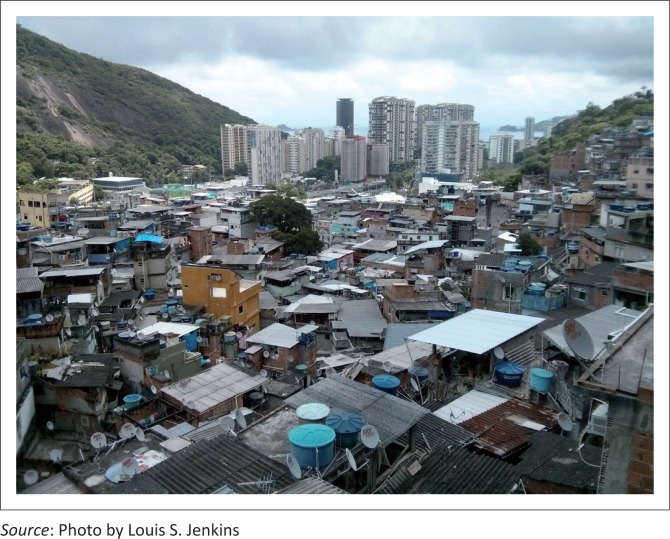
The densely populated suburb of Rocinha favela.

Rocinha (*little farm*) is the largest favela in Brazil, located in Rio de Janeiro’s South Zone between the districts of São Conrado and Gávea. Rocinha is home to approximately 70 000 people (Census 2010), making it the most populous favela in Brazil, a sprawling metropolis of colourful homes, bricks and blocks tied together with crows’ nests of black electric wiring, suspended against the southern slope of a steep hill above the world famous beaches of Ipanema and Leblon. Although Rocinha is officially classified as a neighbourhood, many still refer to it as a favela. It developed from a shanty town into an urbanised slum. Almost all the houses are made from concrete and brick and have basic sanitation, plumbing and electricity. Some buildings are three to four stories tall, and one finds hundreds of businesses such as banks, drugstores, bus lines and cable television (Figures 2 and 3).

A tar road snakes through the area, the main artery flowing with people on motorcycles, on foot, in taxis and on buses. Uncountable alleys branch off the main road, well known to the locals, never entered into by the police. Township order is maintained by the drug dealers. There seems to be a paradoxical peace and sense of safety, with almost no stealing or robberies. Even the use of some illicit drugs, ironically, is severely dealt with by township drug rules. Crack cocaine is produced here, but only does its evil work elsewhere – no one is allowed to use it inside Rocinha.

One of the recent major changes in Rocinha is the presence of the Unidade de Polícia Pacificadora – Pacifying Police Unit (UPP), a new approach to urban safety in the favelas. Historically, police could not enter favelas, as these areas are ruled by the drug dealers. UPP was started in 2008 in another favela, Santa Marta, and in Rocinha in 2011. Policemen stayed in the favelas 24 h a day, 7 days a week, offering continuous community surveillance. Previous police corruption was actively addressed.^12^ Unfortunately, the relationship between the community and the UPP deteriorated in 2013 after a dweller was tortured and killed by the police with the accusation of involvement with drug dealing.^14^

The incidence of tuberculosis in Rocinha is among the highest in Brazil (461/100 000 population) (national surveillance system – SINAN). The southern slope of Rocinha, in particular, never sees the sun in winter. The HIV prevalence (0.46%) is much lower than in South Africa (14%).^2,6^ Although many people living here are working, poverty remains the unwelcome friend of most people, not unlike South Africa where 45% of the population still live on approximately US$2 per day.^6^ Most families now settle for 1–3 children per family.

## Maria do Socorro family clinic

This PHC clinic is the largest of the three that serve the community of Rocinha. The clinic is spacious, light, colourful, well ventilated, with larger than life pictures of patients from the community displayed on the walls as portraits of art. It creates a sense of ownership or agency, and turns the clinic into a kind of home. Adjacent to the clinic is a 24-h walk-in Emergency Department, known as Unidade de Pronto Atendimento (UPA). It has an overnight ward with six short-stay beds, 24-h X-ray facilities, a sonography room (four times a week) and a small onsite laboratory (although most blood tests are processed off site). Patients with surgical and orthopaedic conditions that need specialist care are referred. Patients with the usual conditions that would fall into the ambit of Internal Medicine, like cardiac, renal or neurological disease, are mostly managed locally in the clinic. For example, patients with myocardial infarcts receive alteplase in the clinic and sometimes stay for one to a few days, awaiting transfer to a hospital bed. Patients with diabetic keto-acidosis are mostly managed in the clinic, staying for a few days. This is not necessarily the norm everywhere in Brazil.

On the upper floor, there is also a fantastic mental health facility with six beds, with a resident psychiatrist. Patients who are not coping in the community on their own are identified early by the CHWs and brought here and cared for during the day in a safe communal area, preventing full relapse or complications or risk to the patient or the community. According to Brazilian Mental Health Reform, people that have lived in mental health hospitals and cannot return to their families now have the right to live in the community, with the support of mental health workers, in a Therapeutic Residency. The government funds carers to accompany the patients to their homes and care for them through the night, bringing them back to the mental health unit at the clinic to spend the day there.

South Africa, with an estimated 12-month prevalence of mental illness of 16.5%, has also embarked on de-institutionalising of patients suffering from mental health illness, with the aim of integrating them back into their communities. However, this approach assumes receptive and understanding communities, with a minimum level of care and support, which is often not the case. Subsequently, there has been a national process of integrating mental health into PHC, prioritising the care of patients with mental illness.^15^

A central part of the SUS is the Programa Saúde da Família (PSF), or Family Health Programme. In this clinic, 11 FHTs take responsibility for the care of a specific sub-population of about 2700 people per team in the area that drains to the clinic. Each team works within a well-demarcated boundary, even though there are very few streets. Each team has a doctor and a nursing sister, a nurse assistant and six CHWs. The clinic has 18 family medicine registrars allocated in pairs to 9 teams, bringing the total number of doctors in this clinic to about 30. Furthermore, the teams are supported by a resident psychiatrist, a psychologist, a dietician, two social workers and two physiotherapists. A dermatologist visits every two weeks, and a pneumatologist has a sessional appointment, because of the high tuberculosis burden. There are four dentists onsite, each with a consulting room. The total staff component of the clinic approximates 100. The clinic is managed by two clinic managers, both ex-nursing sisters. Management is stable now, but they went through a difficult time of a high turnover of managers at various levels of competency. The doctor and nursing sister within an FHT share the care of patients. There is not a sense of ‘who is in charge?’ The nursing sister on the team of the second author is busy with her PhD. The relationship is very equal and powerfully collegial. The team knows their patients personally. The second author’s team cares for 40 pregnant women at the time of the first author’s visit. During this visit, the family of a deceased patient came in from the community and a family conference was held, clearly within a relationship of trust and compassion. People know each other – CHWs, nurses, doctors, community and security personnel. Another distinctive aspect of PHC in Brazil is the way the clinic and the community connect, specifically with drug dealers within the community – every clinic has a person who functions as the link between the drug gangs and the health staff, improving communications between the clinic and the community and giving an early warning if things are dangerous.

It seems the South African PHC vision still has a way to go to overcome historical paradigms and power structures and hierarchies, where the doctor almost functioned independently, was often a white male (this is changing in recent years) with a narrow understanding of the health team; the nurses were often female and Black, White, Coloured or Indian; and hospitals were places of healing while clinics and CHWs represented immunisation, malnutrition, contraception and treatment of sexually transmitted diseases. Kautzky and Tollman summarised some of the challenges, including the HIV and AIDS pandemic, health worker shortages and unequal resource distribution, lack of political, public sector and health leadership, and a complex, protracted health transition.^16^ Innovative, experimental work to scale, systems-based approaches and working closely with communities are needed to overcome some of the bureaucratic and rigid thinking of the past, as the country in fact did start out with in the 1940s in Pholela.^17,18,19,20^

## Training

Primary health care vocational training is taken very seriously by the city’s health department. This clinic is a big training centre, with 18 family medicine registrars, 8 PHC nursing students and medical students from different stages of training all working here. Brazil is serious about training doctors and especially family physicians for PHC, which is reflected in their massive improvements in the health outcomes for the country over the last 20 years.^21^ The population of about 200 million people is served by over 200 medical schools, with another 60 in the pipeline, of which half will be rurally situated.^22^ South Africa’s 50 million people are served by nine medical schools, with the 10th medical school opening soon. However, it remains difficult to attract doctors into PHC and family medicine – only 1.2% of the 400 000 doctors in Brazil are trained in Family Medicine.^23^ Despite an increase in the number of training sites each year (total of about 3000 per year), students are not attracted into Family Medicine, with about a 30% occupation rate. For this reason, trainees in Family Medicine are paid more than trainees in traditional specialist training programmes, an initiative governed by various district authorities, such as in Rio de Janeiro. With this incentive, the occupation rate for Family Medicine training in the city is almost 100%. A key driver is high salaries, which will probably become unaffordable in the future, especially with the country’s new austere economic agenda.^24^

The duration of the postgraduate training programme for Family Medicine in Brazil is two years and prepares family physicians for PHC. In South Africa, the training is four years because of the need to function as a generalist expert in a district hospital, which demands a broad spectrum of procedural skills as part of the six roles of the family physician.^25^ The second author is the supervisor of the 18 registrars (trainees) in his clinic and works closely with 9 other tutors. The registrars spend 40 h a week at the PHC clinic and work after hours at the nearby hospital, particularly with the focus on learning about emergencies, paediatrics and obstetrics. This has similarities with the South African model of district hospital training and emphasises the continuity between PHC and emergency care. Very little surgery or orthopaedics are taught, unlike South Africa, where the shortage of district orthopaedic and surgery services necessitates that family physicians have these skills. The clinic has a learning centre with books, furniture and Internet access with resources such as UptoDate© and Dynamed© where students aggregate in between patients to discuss patient problems, ask their supervisor or each other, and learn from each other.

## Conclusion

**FIGURE 4:**
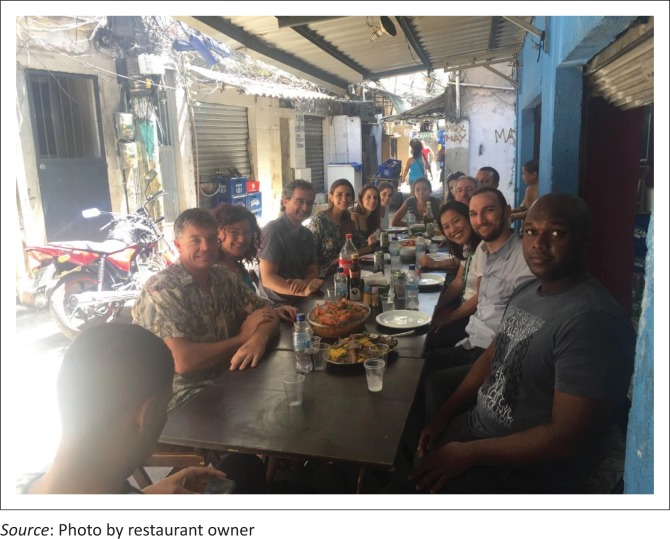
The authors and the health team sharing lunch in Rocinha.

Initially, when the clinic was first opened, the health team was overwhelmed with people with massive health needs and struggled to get through the day. But over time, as the FHT worked in the community, the burden on the clinic lifted. Now it is a very good place to work, with a fine balance of working with and in the community. What was striking is the mutual respect that exists between health cadres in the health team, with the nurses, doctors and security staff all sharing the care of patients, even as they share lunch together (Figure 4). The healthy relationship between the health team and the designated families in the community was very evident in the way the team interacted with young and old, pregnant women, the dying and the mentally ill. Lastly, the integrated way that learning was part and parcel of PHC work, with the many nursing trainees and family medicine registrars in one clinic, and the government incentives to attract trainees to PHC were very enviable. South Africa can do well if political priority is given to PHC as a preferred place for patients and health personnel to find themselves, sharing responsibility for health and learning from each other in community. Being a clinic or community doctor or nurse should become a proud vocation once again.

Parting ways at the bottom of the hill, exiting Rocinha, the first author took the metro, the brand new underground train connecting the people and workers in Rocinha with the suburbs of Ipanema and Copacabana, where some of the most expensive real estate in the southern hemisphere is found, the working and playing grounds of rich and poor. This transport, giving quick, easy access for the people of Rocinha to their working places, as well as for the clinic staff having much improved access from their homes to Rocinha, may be one of the singular most important interventions that made a difference to the health of the community.
